# Species Diversity and Seasonal Abundance of Stomoxyinae (Diptera: Muscidae) and Tabanid Flies (Diptera: Tabanidae) on a Beef Cattle and a Buffalo Farm in Nakhon Si Thammarat Province, Southern Thailand

**DOI:** 10.3390/insects15100818

**Published:** 2024-10-18

**Authors:** Yotsapat Phetcharat, Tuempong Wongtawan, Punpichaya Fungwithaya, Jens Amendt, Narin Sontigun

**Affiliations:** 1 Akkhraratchakumari Veterinary College, Walailak University, Nakhon Si Thammarat 80160, Thailand; panuwat@wu.ac.th (Y.P.); tuempong.wo@mail.wu.ac.th (T.W.); 2 Health Research Center, Walailak University, Nakhon Si Thammarat 80160, Thailand; 3 Center of Excellence Research for Melioidosis and Microorganisms, Walailak University, Nakhon Si Thammarat 80160, Thailand; 4 Office of Administrative Interdisciplinary Program on Agricultural Technology, School of Agricultural Technology, King Mongkut’s Institute of Technology Ladkrabang, Bangkok 10520, Thailand; punpichaya.pu@kmitl.ac.th; 5 Institute of Legal Medicine, University Hospital Frankfurt, Goethe-University, Kennedyallee 104, 60596 Frankfurt am Main, Germany; amendt@em.uni-frankfurt.de

**Keywords:** *Haematobia*, *Stomoxys*, *Tabanus*, vector, flight activity

## Abstract

This study investigated species diversity and seasonal abundance of Stomoxyinae and tabanid flies, which are significant pests and vectors of animal pathogens, on a beef cattle and a buffalo farm in Nakhon Si Thammarat province, southern Thailand. During a one-year period from December 2020 to November 2021, flies were collected using Nzi traps from 6 a.m. to 6 p.m. over three consecutive days each month, resulting in the capture of 1912 biting flies, representing seven Stomoxyinae and nine tabanid species. The five most prevalent species were *Tabanus megalops*, *Haematobia irritans exigua*, *Stomoxys calcitrans*, *Stomoxys indicus*, and *Stomoxys uruma*. Fly density was notably higher on the beef cattle farm compared to the buffalo farm, with most species peaking during the rainy season, except for *H. i. exigua*, which was more abundant during the dry season. This study also examined the influence of temperature, relative humidity, and rainfall on fly density, revealing species-specific patterns. These findings offer updated insights into species diversity and seasonal trends, providing critical baseline data essential for the development of effective control strategies aimed at mitigating the impact of these flies on livestock health.

## Simple Summary

This study investigated species diversity and seasonal abundance of biting flies, specifically Stomoxyinae and tabanid flies, which are important pests capable of spreading diseases to animals, on a beef cattle and a buffalo farm in Nakhon Si Thammarat province, southern Thailand. Over the course of a one-year study (December 2020 to November 2021), 1912 biting flies were collected and identified, comprising seven Stomoxyinae and nine tabanid species. The five most predominant species were *Tabanus megalops*, *Haematobia irritans exigua*, *Stomoxys calcitrans*, *Stomoxys indicus*, and *Stomoxys uruma*. A higher number of flies were collected from the beef cattle farm compared to the buffalo farm, with most species peaking during the rainy season, except for *H. i. exigua*, which was more prevalent during the dry season. Temperature, relative humidity, and rainfall influenced fly density, revealing different patterns depending on the species. This study provides important information that is crucial for designing effective fly control strategies to reduce the impact of these flies on livestock health.

## 1. Introduction

Stomoxyinae flies (Diptera: Muscidae) and tabanid flies (Diptera: Tabanidae) are the two most common blood-sucking insects in many parts of the world that are active and problematic around livestock farms and sometimes attack humans [1,2,3]. Worldwide, there are at least 51 documented species of Stomoxyinae flies in 10 genera [4,5], compared to more than 4400 species of tabanid flies in 156 genera [6]. Both sexes of Stomoxyinae flies and female tabanids of the majority of species are capable of biting and feeding on a wide range of animals (e.g., cattle, horses, dogs, goats, and wild animals). This behavior results in stress, discomfort, irritation, skin lesions, and blood loss, contributing to animal health problems and economic losses in livestock productivity. In addition, they serve as mechanical vectors for numerous pathogens affecting animal and human health, including viruses, bacteria, protozoa, and helminths [1,2]. In terms of the biological vector aspect, *Stomoxys calcitrans* (Linnaeus, 1758) acts as an intermediate host for the stomach worm *Habronema microstoma* in equids. Furthermore, some tabanid flies in the genera *Chrysops* Meigen, 1803, *Haematopota* Meigen, 1803, *Hybomitra* Enderlein, 1922, *Tabanus* Linnaeus, 1758, and *Dasybasis* Macquart, 1847 act as intermediate hosts for filarial nematodes (e.g., *Loa loa*, *Elaeophora schneideri*, *Dirofilaria repens*, and *Dirofilaria roemeri*) and serve as cyclical vectors for *Trypanosoma* (*Megatrypanum*) *theileri* [1,2,7].

In Thailand, 14 species of Stomoxyinae [8] and approximately 128 species of tabanids have been documented [9,10,11]. Stomoxyinae species are distributed across five genera, including *Stomoxys* Geoffroy, 1762 (six species), *Haematobia* Lepeletier & Serville, 1828 (three species), *Haematobosca* Bezzi, 1907 (three species), and one species each of *Haematostoma* Malloch, 1932 and *Stygeromyia* Austen, 1907, with *Stomoxys calcitrans* being the most prevalent and widely distributed [8]. Among tabanids, four genera have been identified—*Tabanus*, *Atylotus* Osten Sacken, 1876, *Chrysops*, and *Haematopota*—with *Tabanus megalops* Walker, 1854, *Tabanus rubidus* (Wiedemann, 1821), and *Tabanus striatus* Fabricius, 1787 being the most frequently observed species on livestock farms [9,10,11,12,13]. Species diversity and seasonal abundance of Stomoxyinae and tabanid flies have been studied in several habitats (e.g., beef cattle farms, dairy farms, buffalo farms, zoos, wildlife conservation areas, and national parks) in several parts of Thailand, including central (e.g., Singburi, Pathumtani, Phra Nakhon Si Ayutthaya, Nakhon Pathom, Chainat, Uthai Thani, Kanchanaburi, and Saraburi), northern (e.g., Mae Hong Son, Chiang Rai, Chiang Mai, and Phitsanulok), northeastern (e.g., Nakhon Ratchasima and Loei), eastern (e.g., Chonburi), and southern (e.g., Chumphon, Prachuap Khiri Khan, Songkhla, Trang, Phatthalung, Nakhon Si Thammarat, and Satun) regions [3,12,13,14]. Although species diversity and seasonal abundance of Stomoxyinae and tabanid flies have been reported in several provinces of southern Thailand, little is known about these flies in Nakhon Si Thammarat province, which ranks among the top five provinces with the largest populations of beef cattle and buffalo in the region.

This study aimed to investigate species diversity and seasonal abundance of Stomoxyinae and tabanid flies on a beef cattle and a buffalo farm in Nakhon Si Thammarat province, southern Thailand, and to analyze their population density in relation to climatic factors, including temperature, relative humidity, and rainfall. The findings of this study will provide essential baseline data for developing effective fly control strategies.

## 2. Materials and Methods

### 2.1. Ethical Approval

This research was approved by Walailak University’s Institutional Animal Care and Use Committee (Approval number: WU-AICUC-63-015).

### 2.2. Study Site and Fly Collection

**Figure 1:**
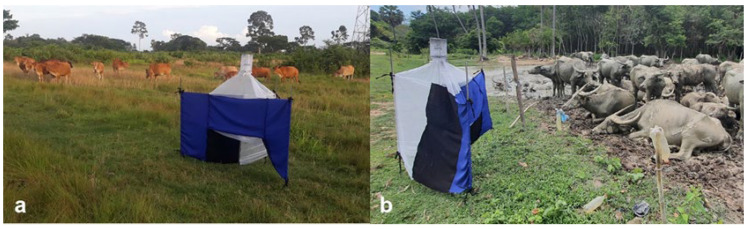
An Nzi trap was placed at a beef cattle farm (**a**) and a buffalo farm (**b**) for fly collection.

Adult Stomoxyinae and tabanid flies were collected over a one-year period, from December 2020 to November 2021, using Nzi traps [15], which are recognized for their effectiveness in capturing diverse species of biting flies. Traps were placed at two locations: a beef cattle farm (N 8°38′40.5672″, E 99°54′07.3296″) and a buffalo farm (N 8°38′16.1016″, E 99°52′27.5016″) in the Tha Sala district, Nakhon Si Thammarat province (Figure 1). Both the beef cattle and the buffalo farm are located within the grounds of Walailak University. The beef cattle farm is situated on a flat plain surrounded by grasslands and perennial vegetation, housing approximately 40 cattle. The buffalo farm, adjacent to a rubber plantation near the Walailak University Botanical Garden, is similarly located on a plain bordered by grasslands and perennial vegetation and houses approximately 70 buffalo. At each farm, one trap was set to capture flies from 6 a.m. to 6 p.m. for three consecutive days each month. Flies were sampled twice daily to prevent decomposition or damage, ensuring accurate identification. On the days of fly collection, a temperature and humidity data logger (ELITECH GSP-6; Elitech Technology Inc., London, UK) was used to record the hourly temperature (°C) and relative humidity (%) at the study sites. The mean monthly rainfall data were obtained from the Thai Meteorological Department. Flies were transported to the Parasitology Laboratory at Akkhraratchakumari Veterinary College, Walailak University.

According to a report by the Thai Meteorological Department, the climate in the southern regions of Thailand is divided into two distinct seasons: the rainy season, which spans from June to January, and the dry season, which lasts from February to May.

### 2.3. Fly Identification

In the laboratory, all collected flies were frozen for 15 min at −40 °C. Only biting flies (Stomoxyinae and tabanid flies) were morphologically identified to the species level using taxonomic keys [4,10,16,17], sexed, and counted under a stereomicroscope (Olympus, Tokyo, Japan). The identified specimens were individually preserved in 1.5 mL microcentrifuge tubes containing 95% ethanol and stored at −40 °C. For Stomoxyinae flies, the right wing of each specimen was carefully excised using fine forceps and placed in a separate 1.5 mL microcentrifuge tube, while the remaining parts of each specimen were preserved as previously described.

### 2.4. Statistical Analysis

The prevalence of each biting fly species was presented in proportions. The diversity indices of the flies collected from each study site were assessed based on the Shannon–Wiener diversity index (H) and Simpson’s diversity index (1-D) using PAST3 software (version 3.09) [18]. The Mann–Whitney U-test was used to compare the differences in fly populations between seasons. Spearman’s rank correlation coefficient (*r_s_*) was used to assess the influence of climatic factors (temperature, relative humidity, and rainfall) on fly density. Statistical analyses were performed using IBM SPSS statistics (version 29.0.0.0) (IBM, Armonk, NY, USA). The statistical significance level was set at *p* < 0.05.

## 3. Results

### 3.1. Species Diversity and Abundance

A total of 1912 biting flies comprising seven species of Stomoxyinae flies and nine species of tabanid flies were collected from the two study sites (Table 1). Among them, the five most abundant species were *Tabanus megalops* (*n* = 683; 35.7%), *Haematobia irritans exigua* (de Meijere, 1903) (*n* = 321; 16.8%), *Stomoxy calcitrans* (*n* = 226; 11.8%), *Stomoxy indicus* Picard, 1908 (*n* = 194; 10.1%), and *Stomoxy uruma* Shinonaga and Kano, 1966 (*n* = 167; 8.7%). *Stomoxy calcitrans* was the most predominant species of Stomoxyinae flies on the beef cattle farm, while *H. i. exigua* was the most abundant on the buffalo farm. For tabanid flies, *T. megalops* was the most prevalent species, followed by *T. rubidus* on both the beef cattle and the buffalo farm. The overall abundance of biting flies was higher on the beef cattle farm (*n =* 1345; 70.3%) compared to the buffalo farm (*n* = 567; 29.7%) (Table 1). Based on the Simpson and Shannon diversity indices, the beef cattle farm demonstrated slightly higher species diversity compared to the buffalo farm, despite the buffalo farm harboring a greater number of insect species (Table 1).

As several biting fly species were found in small numbers (<8% each), they were excluded from the statistical analysis. Therefore, this paper focused on the five most abundant species, including *T. megalops*, *H. i. exigua*, *S. calcitrans*, *S. indicus*, and *S. uruma*. Both male and female Stomoxyinae flies were collected, with more females captured than males, resulting in an overall sex ratio (male/female) of 0.98:1 in *S. calcitrans*, 0.72:1 in *S. indicus*, 0.99:1 in *S. uruma*, and 0.83:1 in *H. i. exigua* (Table 2). With tabanid flies, however, females exclusively were captured.

### 3.2. Seasonal Abundance

**Figure 2:**
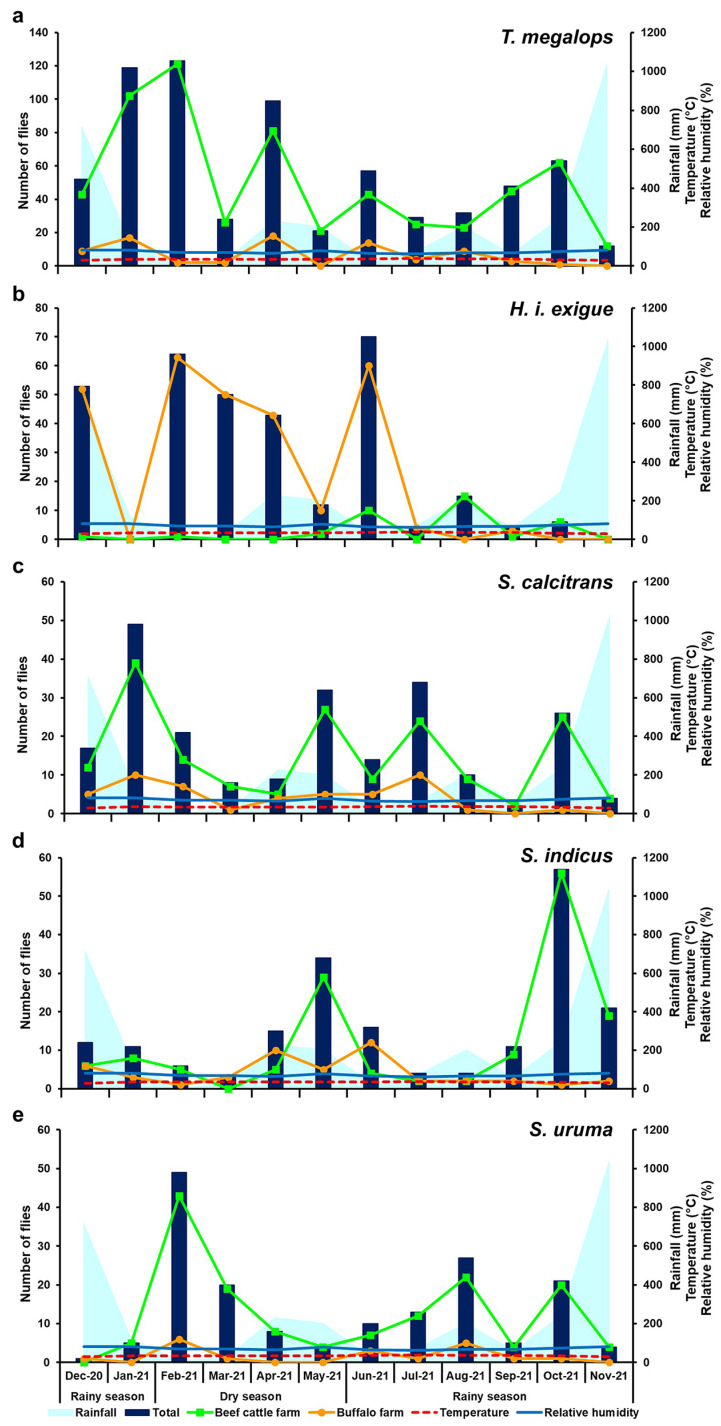
Monthly fluctuations in the total population density of *T. megalops* (**a**), *H. i. exigua* (**b**), *S. calcitrans* (**c**), *S. indicus* (**d**), and *S. uruma* (**e**) trapped in each study site, in relation to the mean temperature and relative humidity recorded at the study sites, along with the mean monthly rainfall data from the Thai Meteorological Department during the study period (December 2020–November 2021).

Seasonal variation in the abundance of the five most prevalent species was determined during two different climatic seasons: the dry season (February to May) and the rainy season (June to January). The overall abundance of biting flies was significantly higher during the rainy season (*n* = 942) compared to the dry season (*n* = 649) (*p* < 0.05) (Table 3). Among the five most prevalent species, although the majority exhibited higher abundance during the rainy season, seasonal differences in fly density were not statistically significant (*p* > 0.05), with the exception of *H. i. exigua*, which was more prevalent in the dry season than in the rainy season (*p* < 0.05) (Table 3). Furthermore, most species were more abundant on the beef cattle farm than on the buffalo farm, whereas *H. i. exigua* was the most prevalent on the buffalo farm (Figure 2).

The fluctuation pattern of *T. megalops* varied throughout the year (Figure 2a), with the highest peak observed at the beginning of the dry season (February 2021). However, the majority of flies (60.3%) were trapped during the rainy season (*n* = 412), with a peak in January 2021, compared to the dry season (*n* = 271; 39.7%). For *H. i. exigua*, higher fly numbers were collected on the buffalo farm compared to the beef cattle farm (Figure 2b). Overall, the greatest number of *H. i. exigua* was trapped during the dry season (*n* = 169; 52.6%), peaking in February 2021, before gradually declining throughout the rest of the dry season. The population then dramatically increased in June 2021 (rainy season), followed by a sharp decrease in July 2021, with low numbers persisting throughout the remainder of the rainy season. The greatest number of *S. calcitrans* was trapped during the rainy season (*n* = 156; 69.0%), with the fluctuation pattern of this species varying throughout the year (Figure 2c), showing three peaks during the rainy season (January, July, and October 2021) and one peak during the dry season (May 2021). Similarly, the greatest number of *S. indicus* was trapped during the rainy season (*n* = 136; 70.1%). Fluctuations were observed throughout the year (Figure 2d), with a major peak in October 2021 (rainy season) and another peak in May 2021 (late dry season). *Stomoxys uruma* numbers were slightly higher during the rainy season (*n* = 86; 51.5%) compared to the dry season (*n* = 81; 48.5%), with fluctuations occurring throughout the year (Figure 2e). The highest trap catch was recorded at the beginning of the dry season in February 2021, followed by a gradual decline. The population then began to increase with the onset of the rainy season, peaking in August 2021 and again in October 2021.

### 3.3. Influence of Climatic Factors on Fly Density

Spearman’s correlation analysis showed no significant relationship between temperature, relative humidity, rainfall, and fly catches of *S. calcitrans* and *T. megalops* (Table 4). The abundance of *S. indicus* was moderately positively correlated with relative humidity (*r_s_* = 0.511, *p* = 0.001) and rainfall (*r_s_* = 0.523, *p* = 0.001), but was moderately negatively correlated with temperature (*r_s_* = −0.438, *p* = 0.008). The abundance of *S. uruma* showed no significant relationship with temperature (*r_s_* = 0.137, *p* = 0.425), but was moderately negatively correlated with relative humidity (*r_s_* = −0.418, *p* = 0.011) and rainfall (*r_s_* = −0.442, *p* = 0.007). In addition, the abundance of *H. i. exigua* was not significantly correlated with temperature (*r_s_* = −0.062, *p* = 0.720) and relative humidity (*r_s_* = −0.204, *p* = 0.232), but was moderately negatively correlated with rainfall (*r_s_* = −0.569, *p* = 0.000)

## 4. Discussion

This study represents the first investigation of the seasonal abundance and species diversity of biting flies, specifically Stomoxyinae and tabanid flies, at a beef cattle and a buffalo farm in Nakhon Si Thammarat province. Over the course of a year, seven species of Stomoxyinae and nine species of tabanid flies were collected. For the *Stomoxys* flies, five out of the six species reported in Thailand were observed in this study, namely *S. bengalensis*, *S. calcitrans*, *S. indicus*, *S. sitiens*, and *S. uruma*. Among the five *Stomoxys* flies found, *S. calcitrans* was the most prevalent species, with 226 specimens (11.8%), and was present throughout the year. This finding is consistent with previous surveys in Thailand that found this species in several habitats (e.g., national parks, wildlife conservation areas, zoos, cattle farms, and dairy farms), particularly on livestock farms [3,19,20]. Furthermore, the abundance of *S. calcitrans* in the rainy season was higher than in the dry season, as revealed in earlier studies [3,19,20]. Regarding other *Stomoxys* flies (i.e., *S. bengalensis*, *S. indicus*, *S. sitiens*, and *S. uruma*), they were found in small numbers depending on the collection sites, which is in agreement with earlier reports [19,20,21]. According to the study by Lorn et al. [3], which investigated the species composition and abundance of *Stomoxys* spp. in Peninsular Thailand (i.e., Songkhla, Trang, Pattalung, Nakhon Si Thammarat, and Satun provinces), they found four *Stomoxys* species, with *S. calcitrans* being the most common species at 87.43%, and fly density was higher during the rainy season. These results are consistent with our study. However, our study found *S. bengalensis* in a beef cattle farm, which did not occur in the dairy and cow farms in the five provinces studied by Lorn et al. [3].

Our study detected only one species of *Haematobia*, namely *H. i. exigua*, commonly known as the buffalo fly, which was more prevalent on the buffalo farm (285 flies) than on the beef cattle farm (36 flies). This finding is consistent with the study by Gudewar et al. [22], which reported a greater fly density on buffaloes than on cattle, suggesting that buffaloes are the primary host for this fly species. Furthermore, this observation is in agreement with earlier studies indicating that buffaloes are the most attractive host for *H. i. exigua* compared to other animals such as zebu, cattle, horses, and dogs [23,24]. Generally, *H. i. exigua* are obligate hematophagous ectoparasites of buffaloes and cattle found in the Australasian, Oriental, and Palaearctic regions of the world, including Thailand [20,25]. This species has also been previously documented in zoos, wildlife conservation areas, and livestock farms, with a particular prevalence in cattle farms [20,21].

Tabanid flies are known to be abundant in a wide variety of habitats, particularly in livestock farms [12,13,26,27]. In this study, *T. megalops* and *T. rubidus* emerged as the two most predominant species on both farms, especially on the beef cattle farm. This finding differs from the study by Changbunjong et al. [12], wherein the predominant species were identified as *T. striatus*, *T. megalops*, and *T. rubidus*, respectively, and were commonly present in villages, primary forests, and secondary forests. The absence of *T. striatus* in our study may be due to the species’ geographical distribution, as it has not been recorded in southern Thailand. However, it has been reported in other regions, including central, western, eastern, northeastern, and northern Thailand [12,13]. In contrast, *T. megalops* and *T. rubidus* are frequently found in villages associated with human activities or settlements [12]. In our study, both the beef cattle and the buffalo farm were located within the grounds of Walailak University, where they are closely associated with human activities. Therefore, it was not surprising that *T. megalops* and *T. rubidus* were the two most predominant species on both farms. In addition, *T. megalops* was found throughout the year, with high abundance during the rainy season, while other species were collected in low numbers. In this study, the abundance of *T. megalops* was higher on the beef cattle farm compared to the buffalo farm, which is located approximately 5 km apart. Notably, despite the presence of another buffalo farm housing approximately 60 buffalo just 1 km from the beef cattle farm, the density of *T. megalops* remained higher at the beef cattle farm. This suggests that beef cattle may be a more attractive host for this fly species.

In this study, the variability in the abundance of *S. indicus*, *S. uruma*, and *H. i. exigua* was found to be influenced by climatic factors, namely temperature, relative humidity, and rainfall, revealing species-specific patterns. Similar results for *S. indicus* were reported by Lorn et al. [3], who conducted a study on a dairy farm in Songkhla. The correlation between *H. i. exigua* density and rainfall observed in this study was inconsistent with the findings of Ngoen-Klan et al. [20], who demonstrated that *H. i. exigua* populations were not significantly influenced by climatic factors. The densities of *S. calcitrans* and *T. megalops* observed in this study were not significantly influenced by climatic factors, consistent with the results reported by Lorn et al. [3] for *S. calcitrans*. However, the findings for *S. calcitrans* in this study contradict previous research [19,20]. Phasuk et al. [19] observed that *S. calcitrans* abundance was significantly correlated with relative humidity and light intensity on dairy farms in Saraburi, while Ngoen-Klan et al. [20] found correlations with rainfall and relative humidity on beef cattle farms in Bangkok. Apart from climatic factors, differences in fly abundance could also be attributed to other variables such as habitat types, altitude, types and quantities of traps used, sunlight exposure on traps, number of collection sites, and duration of the collection period [12,14,28].

Understanding the seasonal abundance of biting flies is essential for developing effective control strategies. The high abundance of biting flies during the rainy season, as observed in this study, suggests that targeting their populations during this period could significantly reduce both the nuisance they cause and the risk of vector-borne diseases impacting livestock health. Additionally, implementing control measures prior to the onset of the rainy season is recommended to prevent fly populations from proliferating.

This study had several limitations that may have impacted the results concerning species diversity and seasonal abundance of flies. Firstly, the number of collection sites was limited; therefore, the overall assessment of species diversity and seasonal abundance of flies may be lower than expected. Secondly, the collection of flies exclusively depended on a single trap type, namely the Nzi trap. This reliance could potentially introduce bias, as the effectiveness of this particular trap may vary for Stomoxyinae and tabanid flies. Nzi traps have demonstrated effectiveness in capturing a diverse range of biting flies [29]. Nonetheless, Vavoua traps have demonstrated effectiveness in collecting *Stomoxys* spp., while Nzi traps were found to be more suitable for capturing tabanid flies [28]. Thirdly, fly collections were conducted only on three consecutive days each month. This limited sampling frequency may affect the observed species diversity and seasonal abundance, possibly resulting in lower estimates than anticipated. To enhance the understanding of the ecological behaviors of biting flies, future studies should include multiple collection sites, utilize diverse types of traps for fly collection, increase the number of traps used at each study site, extend the duration of the collection period, and broaden identification to encompass all hematophagous flies, including non-biting species such as *Musca crassirostris* Stein, 1903.

## 5. Conclusions

Our study provides updated data regarding the species diversity and seasonal abundance of Stomoxyinae and tabanid flies on beef cattle and a buffalo farm in Nakhon Si Thammarat, southern Thailand. With fly populations peaking in the rainy season and declining in the dry season, the risk of vector-borne diseases is likely higher during the rainy season.

## Figures and Tables

**Table 1:** Relative abundance of Stomoxyinae and tabanid flies collected from beef cattle and buffalo farms from December 2020 to November 2021.

Variables	Study Sites		Total Number (%)
	Beef Cattle Farm	Buffalo Farm	
Climatic variable ^a^			
Temperature (°C)	34.2 (28.8–38.0)		
Relative humidity (%)	72.0 (62.6–81.8)		
Rainfall (mm)	245.4 (6.8–1034.6)		
Diversity indices			
Simpson index (1-D)	0.75	0.71	
Shannon index (H)	1.75	1.73	
No. of species	11	16	
Stomoxyinae flies			
*Stomoxys bengalensis* Picard, 1908	93	18	111 (5.8)
*Stomoxys calcitrans* (Linnaeus, 1758)	177	49	226 (11.8)
*Stomoxys indicus* Picard, 1908	145	49	194 (10.1)
*Stomoxys sitiens* Rondani, 1873	70	12	82 (4.3)
*Stomoxys uruma* Shinonaga and Kano, 1966	148	19	167 (8.7)
*Haematobia irritans exigua* (de Meijere, 1903)	36	285	321 (16.8)
*Haematobosca sanguinolenta* (Austen, 1909)	0	5	5 (0.3)
Tabanid flies			
*Tabanus megalops* Walker, 1854	604	79	683 (35.7)
*Tabanus rubidus* (Wiedemann, 1821)	50	28	78 (4.1)
*Tabanus mesogaeus* Peus, 1980	10	7	17 (0.9)
*Tabanus fontinalis* Schuurmans Stekhoven, 1926	0	1	1 (0.1)
*Tabanus virgulatus* Austen, 1922	0	1	1 (0.1)
*Chrysops dispar* (Fabricius, 1798)	10	5	15 (0.8)
*Chrysops flavocinctus* Ricardo, 1902	0	1	1 (0.1)
*Chrysops fuscomarginalis* Burger & Chainey, 2000	2	7	9 (0.5)
*Chrysops fasciatus* Wiedemann, 1821	0	1	1 (0.1)
Total (%)	1345 (70.3)	567 (29.7)	1912 (100)

^a^ Mean (min–max).

**Table 2:** The five most prevalent species collected by Nzi traps from December 2020 to November 2021.

Season	*S. calcitrans*			*S. indicus*			*S. uruma*			*H. i. exigua*			*T. megalops*	Total (%)
	M	F	M/F	M	F	M/F	M	F	M/F	M	F	M/F	F	
Rainy	70	86	0.81	57	79	0.72	42	44	0.96	81	71	1.14	412	942 (59.2)
Dry	42	28	1.5	24	34	0.71	41	40	1.03	65	104	0.63	271	649 (40.8)
Total	112	114	0.98	81	113	0.72	83	84	0.99	146	175	0.83	683	1591

*S. calcitrans* = *Stomoxys calcitrans*, *S. indicus* = *Stomoxys indicus*, *S. uruma* = *Stomoxys uruma*, *H. i. exigua* = *Haematobia irritans exigua*, *T. megalops* = *Tabanus megalops*, M = Male, F = Female, M/F = sex ratio.

**Table 3:** Seasonal abundance of the five most prevalent species collected from December 2020 to November 2021.

Species	Rainy Season	Dry Season	Total (%)	*p*-Value
*T. megalops*	412	271	683 (42.9%)	0.311
*H. i. exigua*	152	169	321 (20.2%)	0.001
*S. calcitrans*	156	70	226 (14.2%)	0.753
*S. indicus*	136	58	194 (12.2%)	0.068
*S. uruma*	86	81	167 (10.5%)	0.137
Total	942	649	1591 (100%)	0.020

*S. calcitrans* = *Stomoxys calcitrans*, *S. indicus* = *Stomoxys indicus*, *S. uruma* = *Stomoxys uruma*, *H. i. exigua* = *Haematobia irritans exigua*, *T. megalops* = *Tabanus megalops*.

**Table 4:** Spearman’s rank correlation coefficient values between the climatic factors and fly populations collected from December 2020 to November 2021.

Climatic Factors		Species				
		*S. calcitrans*	*S. indicus*	*S. uruma*	*H. i. exigua*	*T. megalops*
Temperature	*r_s_*	−0.059	−0.438	0.137	−0.062	0.040
	*p*	0.731	0.008	0.425	0.720	0.819
Relative humidity	*r_s_*	0.111	0.511	−0.418	−0.204	−0.126
	*p*	0.518	0.001	0.011	0.232	0.465
Rainfall	*r_s_*	0.106	0.523	−0.442	−0.569	−0.305
	*p*	0.539	0.001	0.007	0.000	0.071

*S. calcitrans* = *Stomoxys calcitrans*, *S. indicus* = *Stomoxys indicus*, *S. uruma* = *Stomoxys uruma*, *H. i. exigua* = *Haematobia irritans exigua*, *T. megalops* = *Tabanus megalops*.

## Data Availability

The data presented in the study are available in the article.
